# Preliminary Evaluation of a Recombinant Rift Valley Fever Virus Glycoprotein Subunit Vaccine Providing Full Protection against Heterologous Virulent Challenge in Cattle

**DOI:** 10.3390/vaccines9070748

**Published:** 2021-07-06

**Authors:** William C. Wilson, Bonto Faburay, Jessie D. Trujillo, Izabela Ragan, Sun-Young Sunwoo, Igor Morozov, Vinay Shivanna, Aaron Balogh, Kinga Urbaniak, D. Scott McVey, Dashzeveg Bold, Natasha N. Gaudreault, Erin E. Schirtzinger, Wenjun Ma, Juergen A. Richt

**Affiliations:** 1 United States Department of Agriculture, Agricultural Research Service, Arthropod Borne Animal Disease Research Unit, Manhattan, KS 66502, USA; dmcvey2@unl.edu (D.S.M.); eesem@vet.k-state.edu (E.E.S.); 2 Department of Diagnostic Medicine/Pathobiology, College of Veterinary Medicine, Kansas State University, Manhattan, KS 66506, USA; Bonto.Faburay@usda.gov (B.F.); jdtrujillo@vet.k-state.edu (J.D.T.); Izabela.Ragan@colostate.edu (I.R.); sunwoosy@gmail.com (S.-Y.S.); imorozov@vet.k-state.edu (I.M.); vshivanna@txbiomed.org (V.S.); aaron.balogh@wisc.edu (A.B.); kinga.urbaniak@piwet.pulawy.pl (K.U.); bold@vet.k-state.edu (D.B.); nng5757@k-state.edu (N.N.G.); wma@missouri.edu (W.M.)

**Keywords:** Rift Valley fever virus, cattle, subunit vaccine, efficacy

## Abstract

Rift Valley fever virus (RVFV) is a mosquito-borne zoonotic pathogen that causes periodic outbreaks of abortion in ruminant species and hemorrhagic disease in humans in sub-Saharan Africa. These outbreaks have a significant impact on veterinary and public health. Its introduction to the Arabian Peninsula in 2003 raised concerns of further spread of this transboundary pathogen to non-endemic areas. These concerns are supported by the presence of competent vectors in many non-endemic countries. There is no licensed RVF vaccine available for humans and only a conditionally licensed veterinary vaccine available in the United States. Currently employed modified live attenuated virus vaccines in endemic countries lack the ability for differentiating infected from vaccinated animals (DIVA). Previously, the efficacy of a recombinant subunit vaccine based on the RVFV Gn and Gc glycoproteins, derived from the 1977 human RVFV isolate ZH548, was demonstrated in sheep. In the current study, cattle were vaccinated subcutaneously with the Gn only, or Gn and Gc combined, with either one or two doses of the vaccine and then subjected to heterologous virus challenge with the virulent Kenya-128B-15 RVFV strain, isolated from *Aedes* mosquitoes in 2006. The elicited immune responses by some vaccine formulations (one or two vaccinations) conferred complete protection from RVF within 35 days after the first vaccination. Vaccines given 35 days prior to RVFV challenge prevented viremia, fever and RVFV-associated histopathological lesions. This study indicates that a recombinant RVFV glycoprotein-based subunit vaccine platform is able to prevent and control RVFV infections in target animals.

## 1. Introduction

The emergence or re-emergence of vector-borne diseases is of increasing global concern [1,2]. Rift Valley fever (RVF) is one of these vector-borne diseases raising serious concerns for its potential introduction into Europe and the United States (US), since competent mosquito vectors exist on both continents [3,4,5]. The RVF virus (RVFV) is classified as an overlap select agent and risk group-3 pathogen by the Centers for Disease Control and Prevention (CDC) and the United States Department of Agriculture (USDA), because of its potential veterinary and public health impacts. Primary control measures to prevent further spread of RVFV, especially during outbreaks, relies on the efficient use of efficacious veterinary vaccines. Although the safety of an investigational inactivated human RVF vaccine has been described, this vaccine requires multiple vaccinations to elicit protective neutralizing antibody titers [6]. In addition, there is also a modified live attenuated RVF vaccine candidate in Phase II clinical evaluation [7,8]; however, neither of the human vaccines are readily available or licensed. There are veterinary RVF vaccines available for RVF endemic countries but only one veterinary vaccine is conditionally licensed in the U.S. (reviewed [9]). Although the modified live attenuated virus vaccines available in endemic countries are efficacious [10,11], safety issues associated with their use in non-endemic regions remain a major concern [12,13]. The modified live attenuated vaccines also do not readily support differentiating infected from vaccinated animals (DIVA), which is a preferred prerequisite for state-of-the-art animal vaccines to control and eradicate infectious diseases upon introduction into non-endemic areas.

A member of the *Phenuiviridae* family, RVFV has a tripartite single-stranded negative RNA genome composed of small (S), medium (M) and large (L) RNA segments. The S segment encodes the nucleocapsid protein (N) and the non-structural protein NSs. The M segment encodes the two glycoproteins, Gn and Gc, the 78-kDa protein and the non-structural protein, NSm. The L-segment encodes the RNA-dependent RNA polymerase [14]. The surface glycoproteins, Gn and Gc, contain epitopes that elicit the induction of neutralizing antibodies, the only established correlates of protective immunity against RVFV infection [15,16,17,18]. These findings provided the foundation for development of a subunit vaccine based on Gn and Gc [15]. Currently licensed vaccines are either inactivated or modified live attenuated vaccines. Only one of these vaccines, namely the NSs-deleted attenuated virus vaccine, Clone 13, is potentially DIVA compatible; however, the immune response to NSs in naturally infected ruminants is inconsistent [19], which makes using the NSs protein as a DIVA marker of limited value. In addition, RVF DNA vaccines have also been developed [20] but these are expensive and difficult to deliver in endemic areas. In contrast, a subunit DIVA-compatible vaccine platform provides a safe approach for production, scale-up, distribution and use. In previous studies, we have described the development of a recombinant RVFV Gn/Gc subunit vaccine and demonstrated its efficacy in a target animal species, sheep [21]. Preliminary mouse studies indicated that Gn alone could produce an immune response that was protective (unpublished data). Here, we performed preliminary evaluation of the efficacy using various formulations and application strategies of the recombinant subunit vaccine to protect cattle against virulent RVFV challenge.

## 2. Materials and Methods

### 2.1. Ethics Statement

All animal studies were carried out in accordance with guidelines set forth by the Animal Welfare Act, The Guide for the Care and Use of Laboratory Animals, 8th edition and/or The Guide for the Care and Use of Agricultural Animals in Research and Teaching, 3rd edition, as applicable for each species. The Kansas State University Institutional Biosafety (IBC) and Animal Care and Use Committees (IACUC) approved and provided oversight for this study. The experimental work described herein falls under KSU IBC protocol #1004, and IACUC protocols #3518.

### 2.2. Viruses and Cells

The RVFV Kenya 2006-128b-15 (Ken06) [22] isolate was provided by R. Bowen, Colorado State University, Fort Collins, CO, through B. Miller, Centers for Disease Control, Fort Collins, CO. The Ken06 virus strain was propagated in a C6/36 *Aedes albopictus* cell line (ATCC, Manassas, VA, USA) with MEM culture medium (Life Technologies, Grand Island, NY, USA), supplemented with 10% fetal bovine serum (FBS; Sigma-Aldrich, St. Louis, MO, USA) and 1× Penicillin/Streptomycin/Fungizone (PSF; Gibco, Grand Island, NY, USA). The *A. albopictus* cell line was maintained at 28 °C, whereas virus-infected insect cells were maintained at 37 °C. MP-12 is a non-virulent strain of RVFV, attenuated via chemical mutagenesis [23], and was used as the viral stock in plaque reduction neutralization assays [21]. Vero MARU (Middle America Research Unit, Panama) cells were used for virus isolation and titration. The cells were grown in Medium M-199 (M199E) culture medium (Sigma-Aldrich), supplemented with 10% FBS and 1× PSF, and maintained in a 37 °C, 5% CO_2_ incubator.

### 2.3. Recombinant Baculovirus Expression and Purification of RVFV Gn and Gc Glycoproteins

The cloning and creation of the recombinant baculovirus constructs for expression of RVFV glycoproteins Gn and Gc has been described previously [15,24]. The ectodomain of the Gn glycoprotein (Gne) was expressed, which hereafter will be referred to as Gn. The Gc glycoprotein was expressed as a full-length protein. Recombinant protein expression was carried out as described previously [21]. Aliquots of the protein were stored at −80 °C until use.

### 2.4. Vaccine Preparation

To prepare the subunit vaccine, recombinant Gn or recombinant Gn and Gc glycoproteins were formulated in Montanide^TM^ ISA-25 VG (Seppic, France), a ready-to-use vaccine adjuvant for oil-in-water (O/W) emulsion, to obtain 50 µg of each antigen (Gn and Gc) per vaccine dose according to the manufacturer’s recommendation.

### 2.5. Animals, Vaccination and Viral Challenge

**Figure 1:**
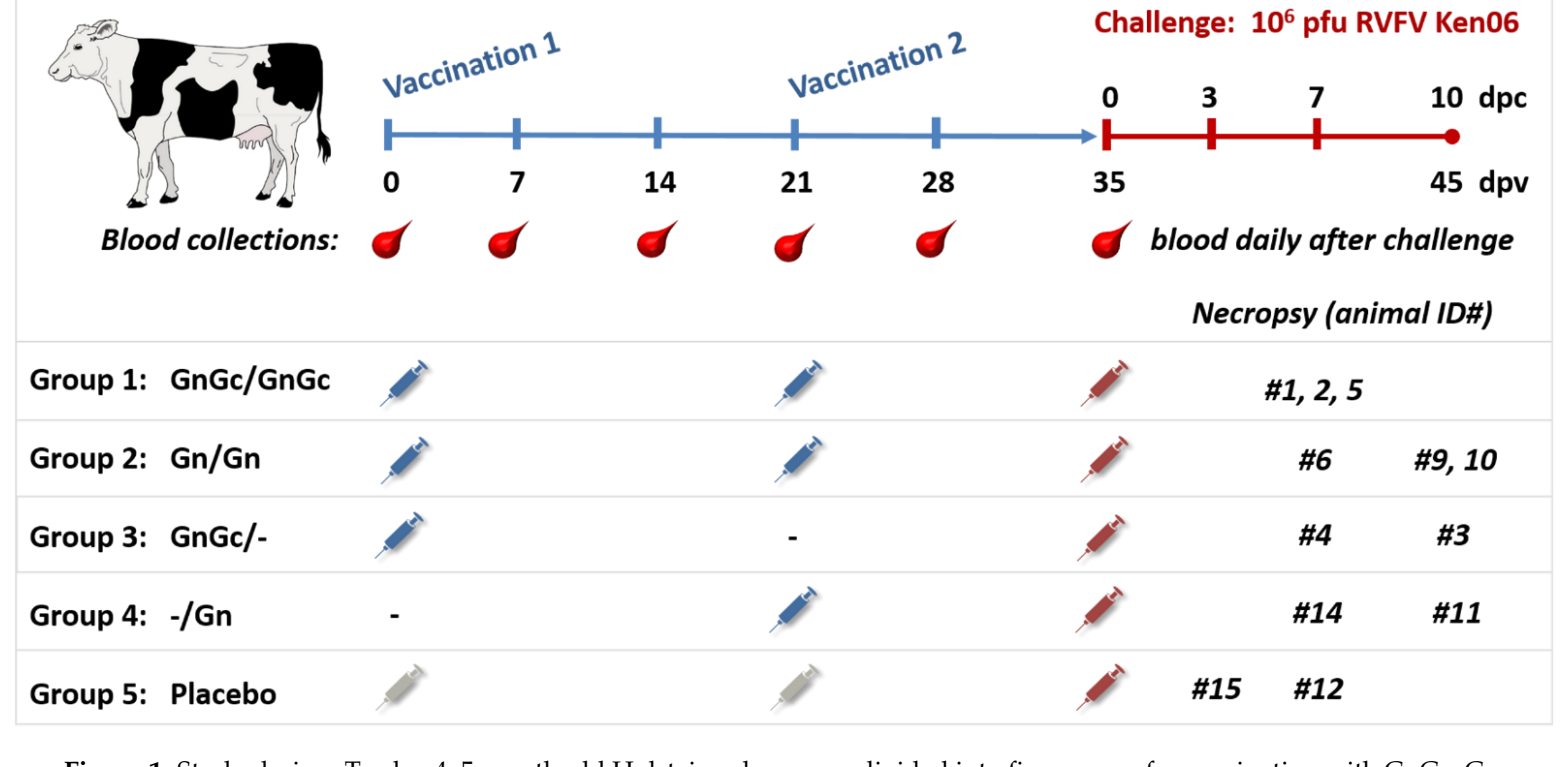
Study design. Twelve 4–5-month-old Holstein calves were divided into five groups for vaccination with GnGc, Gn only, or adjuvant only (placebo group). Calves were vaccinated subcutaneously at 0 and/or 21 days as indicated. At 35 days post first vaccination (dpv), calves were challenged with RVFV subcutaneously. Calf #15 died with acute RFV, and was necropsied at 3 days post challenge (dpc); the remaining calves were necropsied at 7 dpc and 10 dpc as indicated.

Twelve naïve healthy cattle (*Holstein Friesian breed*), aged 4–5 months, were obtained from a private breeder in Kansas, USA. The calves were acclimated for seven days at the Large Animal Research Center (LARC; Kansas State University, Manhattan, KS, USA) and subjected to deworming using Draxxin^®^ (Zoetis) and Albendazole. The animals were divided into five groups (Figure 1). Each treatment was administered using a 2 mL subcutaneous inoculation. Group 1 (N = 3; animals #1, 2 and 5) were administered 50 µg each of the Gn and Gc glycoproteins at 0 and 21 days post vaccination (dpv). Animals in group 2 (N = 3; animals #6, 9 and 10) were administered 50 µg of the glycoprotein Gn only at 0 and 21 dpv. Group 3 (N = 2; animals #3 and 4) were administered 50 µg of each of the glycoproteins Gn and Gc at 0 dpv only. Group 4 (N = 2; animals #11 and 14) were administered 50 µg of Gn at 21 dpv only. Group 5 (N = 2 animals #12 and 15) served as mock-vaccinated, virus challenge controls, and were administered with an equivalent volume of adjuvant only at 0 and 21 dpv. Pre-vaccination blood samples were collected from all animals at 0 dpv, and weekly thereafter from 7 to 35 dpv. The animals were monitored during the first three dpv for changes in rectal temperature and localized inflammation at the site of vaccine administration. Additionally, the vaccination sites were monitored during a 35-day post-vaccination period for occurrence of erythema, tissue nodules or abscess formation. At 28 dpv, the animals were relocated from the LARC to the Biosecurity Research Institute (BRI), which is the BSL-3Ag facility at Kansas State University. To assess the protective efficacy of the vaccine, at 35 dpv, corresponding to 0 days post challenge (dpc), all animals were challenged subcutaneously with 2 mL of 1 × 10^6^ plaque forming units (pfu) of the Ken06 RVFV strain. Post-challenge, all animals were monitored daily for clinical signs, including rectal temperature. Blood samples for virological, immunological and blood chemistry analyses were collected daily from 0 to 7 dpc. Necropsies were performed and tissue samples were collected for histopathology from all animals, including animal #15, which died 3 days post challenge, from animals #1, 2, 4, 5, 6, 12 and 14 at 7 dpc, and from animals #3, 9, 10, and 11 at 10 dpc.

### 2.6. Viral RNA Extraction and Real-Time RT-qPCR

Total RNA was extracted from serum using TRIzol-LS reagent (Life Technologies) as described previously [21]. Aqueous phase (100 μL) was added to 500 μL of Lysis buffer (GeneReach USA) and subjected to magnetic bead extraction using the total nucleic acid (NA) magnetic bead-based extraction kit (GeneReach USA) with modifications. Modifications include the substitution of 100% molecular grade isopropanol for ethanol, which is added to the lysate after the addition of lysates to magnetic beads, and the use of 100% molecular grade ethanol as the final wash solution. Extractions were performed on the automated bead processor (Biosprint, Qiagen, Germany or Taco Mini, GeneReach USA) and RNA was eluted into 100 μL of elution buffer.

For RT-qPCR performed on formalin fixed paraffin-embedded liver tissue, paraffin scrolls (N = 10; 5 μm thick) were placed into 320 μL deparaffinization solution (Qiagen, Hilden, Germany), vortexed and incubated at 56 °C for 5 min, then cooled to room temperature. A total of 80 μL of alkaline tissue lysis buffer (Qiagen) was added. The lysate was vortexed and centrifuged for 1 min at 10,000 rpm. Then, 40 μL of proteinase K (Qiagen) was added to the lower phase and the sample was incubated at 56 °C for 30 min, followed by incubation at 80 °C for 30 min. The lower phase was transferred to 320 μL RLT buffer (Qiagen) and vortexed. Finally, 200 μL of the lysate was processed using the magnetic bead extraction protocol as described above.

A published triplex real-time reverse transcriptase-polymerase chain reaction (RT-qPCR) assay was used to detect each of the three RVFV RNA genome segments [25], using qScript XLT one-step RT-qPCR ToughMix (Quanta Biosciences) and 2.5–5 μL of RNA into a 20 μL RT-qPCR reaction, performed on the CFX 96 real time PCR machine (Biorad). The cut-off cycle threshold (Ct) value was set at 35 for each gene segment.

For quantitative RT-qPCR, in vitro transcribed RNA (IVT RNA) was generated using the T7 transcription kit (MEGAscript, ThermoFisher) and RT-PCR-generated cDNA. RNA was generated from plasmids containing partial RVFV L, M and S gene sequences, SuperMix (Quanta Biosciences) and T7 promoter and terminator primers (Integrated Technologies). IVT RNA was DNAse treated 3 times, column purified (MEGAclear, ThermoFisher) and quantitated with spectrophotometry. The RNA copy number (CN) was calculated using an online calculator system (http://scienceprimer.com/copy-number-calculator-forrealtime-pcr, accessed in 2017). Tenfold serial dilutions of IVT RNA stock (10^4^ to 10^−1^ RNA copies) were utilized to generate a six-point standard curve using six RT-qPCR well replicates per dilution for each RVFV gene segment. RNA CN for samples were mathematically determined using the RT-qPCR determined mean Ct for the L, M and S segments for the respective samples tested, and the slope and intercept of the respective gene segment IVT RNA reference standard curve. Data are reported as RT-qPCR determined RNA CN per ml. Calculated RNA CN less than 15 (equivalent to a Ct greater than 36) are considered below limit of detection (LOD) for the RT-qPCR assay and are classified as equivocal and thus are not reported as true positives.

### 2.7. Virus Titration

Virus challenge material, cattle liver and cattle sera were titrated by standard plaque assay on Vero MARU cells. Briefly, confluent cell monolayers were inoculated with ten-fold serially diluted samples in M199E and incubated for 1 h. Following adsorption, the inocula were replaced with a 1:1 mixture of 2% carboxymethyl cellulose (Sigma-Aldrich) in 2x M199E (20% FBS and 2x PSF) and returned to the incubator. After 5 days, cells were fixed and stained with crystal violet fixative (25% formaldehyde, 10% ethanol, 5% acetic acid, 1% crystal violet). Virus plaques were counted to determine the pfu/mL.

### 2.8. Serology and Blood Chemistry

#### 2.8.1. Immunogen-Specific Indirect ELISA

Vaccine-induced seroconversion was monitored at 0, 28 and 35 dpv. For this purpose, anti-RVFV Gn-specific antibodies were detected using the indirect ELISA method described previously [15,26]. The cut-off point for seroconversion was determined for each individual animal and was determined by adding three standard deviations to the corresponding mean OD value of the pre-vaccination serum. Mean OD values equal to or greater than the cut-off value were considered positive.

#### 2.8.2. Fluorescence Microsphere Immunoassay (FMIA)

Anti-RVFV Gn and nucleoprotein (N) antibodies were detected simultaneously using a fluorescence microsphere immunoassay (FMIA) as previously described [27]. The assay median fluorescent intensity (MFI) cutoff values for bead targets were: 2500 MFI for the N target and 3800 for Gn target. Plates were analyzed on the Luminex MAGPIX^®^ System using xPONENT version 4.2 software (Luminex Corporation, Austin, TX, USA).

#### 2.8.3. Plaque Reduction Neutralization Test (PRNT)

Assessment of anti-RVFV neutralizing antibody responses to vaccination was performed using the plaque reduction neutralization test (PRNT_80_) using MP12 RVFV as described previously [15,21].

#### 2.8.4. Blood Chemistry Analysis

Serum blood chemistry analysis was performed using a VetScan VS2 Chemical Analyzer and the Large Animal Profile rotor (Abaxis, Union City, CA, USA) as described by the manufacturer. The VetScan Large Animal Profile reagent rotor provides quantitative determinations of albumin (ALB), alkaline phosphatase (ALP), aspartate aminotransferase (AST), calcium (CA++), creatine kinase (CK), gamma glutamyl transferase (GGT), globulin (GLOB), magnesium (MG), inorganic phosphorus (PHOS), total protein (TP) and urea nitrogen (BUN) in heparinized whole blood, heparinized plasma or serum.

### 2.9. Pathology

All animals enrolled in the study were humanely euthanized and necropsied. The following tissues were collected at necropsy from all animals and placed in 10% neutral buffered formalin for at least 7 days before further processing: liver, spleen, kidney, adrenal gland, mesenteric lymph node, lung and eye. These samples were trimmed, placed in cassettes, dehydrated and embedded in paraffin. Four µm thick tissue sections were cut and placed onto positively charged glass slides for hematoxylin-and-eosin (H&E) staining and immunohistochemistry (IHC; see below). The slides were examined and scored by a veterinary pathologist in a blinded fashion. Liver was scored on a scale from 0–4 as described previously [28]. Briefly, liver scores of 0 are essentially normal with minimal to no portal tract infiltrates. Scores of 1 include the presence of small aggregates or low numbers of inflammatory cells within hepatic lobules that can be associated with minimal hepatocyte degeneration/necrosis, and these scores can be considered background or a result of a mild infection when associated with RVFV viremia. Liver scores equal to or greater than 2 are associated with more extensive hepatic necrosis/inflammation and/or hemorrhage; these are attributed to RVFV infection and are confirmed when associated with viremia and/or presence of infectious virus, virus antigen or viral RNA within the lesions/liver.

Immunohistochemistry for the detection of RVFV antigen in tissues was conducted as described previously [21,28]. Briefly, slides were deparaffinized, rehydrated and antigen was retrieved using a vegetable steamer technique in pH 6.0 citrate buffer with detergents (DAKO; Carpinteria, CA, USA) for 20 min. Between all steps, tissue sections were washed in Tris-buffered saline 1× (TBS) with 0.01% tween-20 (TBSt). The slides were incubated with 3% hydrogen peroxide and serum blocked per manufacturer instructions (VECTASTAIN Elite ABC-HRP Kit, Peroxidase (Rabbit IgG)–(PK-6101), Vector Labs (VL); Burlingame, CA, USA). Tissues were briefly rinsed in TBSt then incubated overnight at 4 °C with the primary antibody, a polyclonal rabbit anti-RVFV nucleoprotein antibody [28], diluted 1:500 in TBS. Post primary antibody incubation, a matched secondary antibody and avidin–biotin complex detection reagents were applied per manufacturer’s instructions (PK-6101, VL). Antigen was visualized with 3′3-diaminobenzidine (DAB) chromogen followed by a Mayer’s hematoxylin counterstain. Image capture and post-processing of histopathology was conducted as described previously [28].

### 2.10. Statistical Analysis

Analyses for statistically significant differences between vaccinated and control animals were performed for rectal temperatures as well as for serum AST and BUN values. Due to large variation of values among individual animals, the geometric mean values were derived at each time point. Group mean values of rectal temperature responses for vaccinated and non-vaccinated groups per time point were also determined. A paired t-test was performed to analyze AST values, whereas grouped analysis using a two-way ANOVA was performed to analyze rectal temperature and BUN values. The FMIA, ELISA, RT-qPCR, virus titer and PRNT80 data were analyzed using a two-way ANOVA analysis, followed by a post hoc Turkey’s *t*-test, to determine significant differences between groups reported as adjusted p values. Analysis was done using GraphPad Prism software (version 6 and 8.1.1) (GraphPad Software, Inc., La Jolla, CA, USA).

## 3. Results

### 3.1. Immunogenicity of the Gn and the GnGc RVF Vaccines

**Figure 2:**
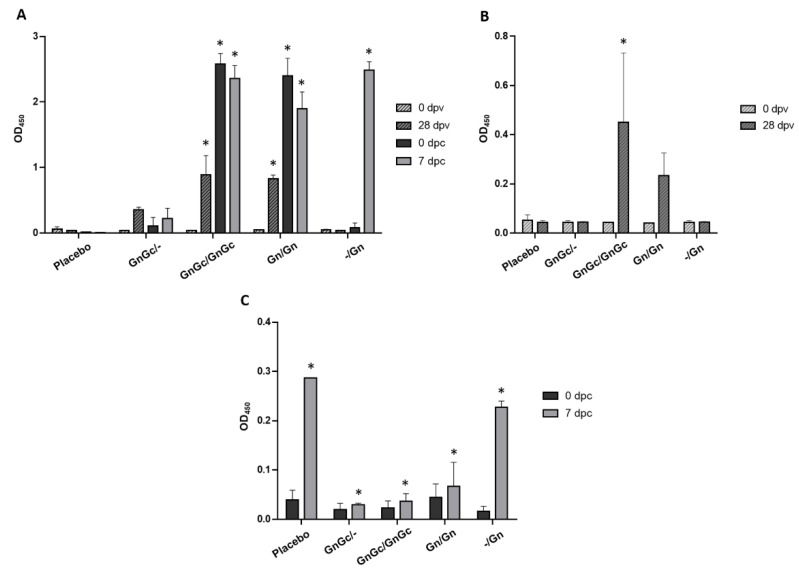
Indirect Enzyme Linked Immunosorbent Assay (ELISA) determination of RVFV antibody responses of vaccinated and infected cattle using recombinant RVFV (**A**) Gn, (**B**) Gc and (**C**) N antigens. Antibody responses of cattle to candidate RVFV subunit vaccines containing Gn with Gc or Gn alone, with or without a booster vaccination, were evaluated. Antibody responses against RVFV N protein was used to distinguish vaccinated from infected cattle. The detection of IgG antibodies in sera at 0 and 28 days post vaccination (dpv) and 0 and 7 days post challenge (dpc) are shown; 0 and 7 dpc are equal to 35 and 42 dpv, respectively. The detection of antibodies is reported as mean Optical Density (OD) at 450 nm. Significance was determined by two-way ANOVA with Tukey’s multiple comparisons test for adjusted p values using Graph Pad Prism software. (**A**) At 28 dpv, GnGc/GnGc and Gn/Gn groups were significantly different (* *p* value < 0.0001) compared to the placebo and -/Gn groups, and the GnGc/GnGc and Gn/Gn groups were significantly different (* *p* value < 0.01 and < 0.05, respectively) compared to the GnGc/- group; at 0 dpc, the GnGc/GnGc and Gn/Gn groups were significantly different (* *p* value < 0.0001) compared to the placebo, GnGc/- and -/Gn groups; at 7 dpc, the GnGc/GnGc, Gn/Gn and -/Gn groups were significantly different (* *p* value < 0.0001) compared to the placebo and GnGc/- groups, and the Gn/Gn group was significantly different (* *p* value < 0.01) compared to the GnGc/GnGc and -/Gn groups. (**B**) The GnGc/GnGc group was significantly different (* *p* value < 0.05) compared to the placebo, GnGc/- and -/Gn groups at 28 dpv. (**C**) At 7 dpc, GnGc/GnGc, Gn/Gn and GnGc/- groups were significantly different (* *p* value < 0.0001) compared to the placebo and -/Gn groups.

The US-raised cattle did not exhibit any background antibody response to the recombinant RVFV Gn or Gc glycoproteins prior to vaccination at day 0 (Figure 2A,B). By 28 days post vaccination (dpv), all Gn/Gn vaccinated animals developed a detectable anti-Gn antibody response, which greatly improved after the booster vaccination at 35 dpv (0 dpc) (Figure 2A). The animals that only received one Gn vaccination at 21 dpv did not develop a detectable anti-Gn antibody response when tested at 28 dpv or 35 dpv/0 dpc (Figure 2A). The animals that received the combined Gn/Gc vaccine at 0 and 21 dpv had a low-level antibody response to Gc at 28 dpv, but no response to Gc was detected in animals that only received one dose of the GnGc vaccine at 0 dpv (Figure 2B). Cattle immunized twice with the Gn only vaccine at 0 and 21 dpv had increased background antibody activity against the Gc in the ELISA (Figure 2B), whereas the RVFV N-specific ELISA demonstrated no detectable antibodies in these animals at 35 dpv/0 dpc, prior to challenge (Figure 2C). Post challenge, groups 1–3 had only a slight increase in antibodies to N compared to the placebo or -/Gn group (Figure 2C).

**Figure 3:**
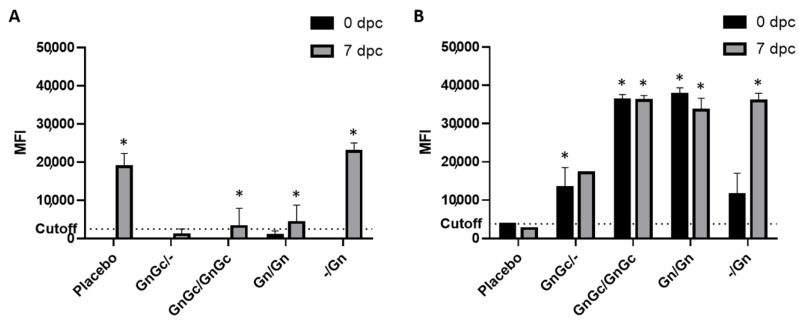
RVFV Fluorescence Microsphere Immunoassay FMIA enables differentiation of infected from vaccinated (DIVA) cattle using (**A**) RVFV N and (**B**) Gn recombinant proteins. FMIA was used to further evaluate the efficacy of candidate RVFV subunit vaccines to induce antigen-specific antibodies. The cattle were vaccinated with the respective subunit vaccine formulations and at 35 days post initial vaccination were challenged with the RVFV Ken06 strain and maintained for up to 10 days post challenge (dpc). The detection of IgG antibodies in sera against the N and the Gn target proteins at 0 and 7 dpc are shown, and reported as median fluorescence intensity (MFI). Assay cutoff is at 2500 MFI for the N target and 3800 MFI for the Gn target. RVFV N served as the DIVA-compatible marker. Significance was determined by two-way ANOVA with Tukey’s multiple comparisons test for adjusted p values using Graph Pad Prism software. (**A**) At 7 dpc, GnGc/GnGc, GnGc/- and Gn/Gn groups were significantly different (* *p* value < 0.001) compared to the placebo and -/Gn groups. (**B**) At 0 dpc, GnGc/GnGc and Gn/Gn groups were significantly different (* *p* value < 0.0001) compared to the placebo, GnGc/- and -/Gn groups and GnGc/- groups were significantly different (* *p* value < 0.05) compared to placebo; at 7 dpc, GnGc/GnGc, Gn/Gn and -/Gn groups were significantly different (* *p* value < 0.0001) compared to the placebo, and the GnGc/GnGc group versus the GnGc/- group; also the GnGc/- group was significantly different (* *p* value < 0.001) compared to the Gn/Gn and -/Gn groups.

FMIA of the cattle sera demonstrated the DIVA compatibility of the RVFV subunit vaccine candidates. The FMIA joint analysis of antibody responses to the N, Gn and Gc target proteins was able to differentiate vaccinated groups from the placebo group (Figure 3). After vaccination, but before challenge with virulent RVFV at 35 dpv/0 dpc, the placebo group had no detectable antibodies against N (Figure 3A) or Gn (Figure 3B). All Gn or GnGc vaccination groups had detectable antibodies against the Gn target and no detectable antibodies against the N target. The GnGc and Gn-only animals that received a second dose of vaccination had a FMIA signal only against the Gn and not the N target and the difference was statistically significant (*p* value < 0.05) when compared to the placebo group. Seven days after challenge (7 dpc), the placebo group had an increase in detectable antibodies against the N target but no increase was noted for the Gn target. All vaccination groups had increased MFI signals to N post challenge, but only the late single dose GnGc and Gn vaccine groups had MFI signals significantly above the assay cutoff against the N target (Figure 3). All vaccination groups had MFI signals above the assay cutoff against the Gn target, and the differences were statistically significant (*p* value < 0.05) when compared to the placebo group. Except for the group vaccinated once with Gn at 21 dpv, all vaccinated groups reported minimal MFI signal for antibodies to N and high MFI signal for antibodies to Gn after challenge. The differences in the MFI signals between these two targets support the DIVA capability of the Gn and GnGc vaccine candidates.

### 3.2. Efficacy of the RVFV Vaccine Candidates

**Figure 4:**
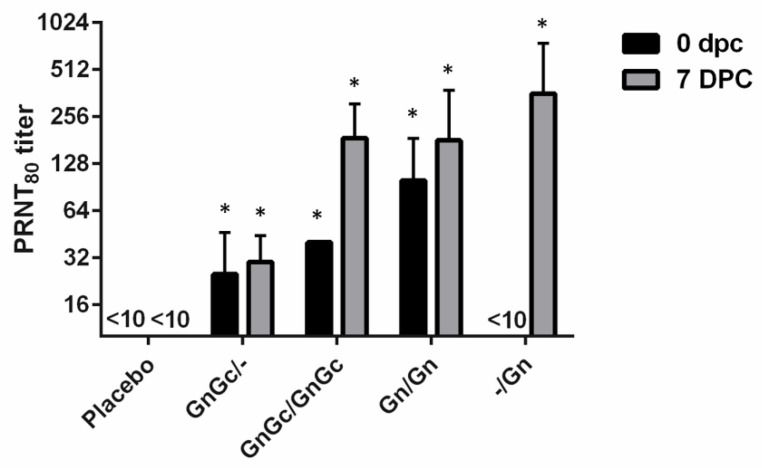
RVFV plaque reduction neutralization assay of 80% (PRNT_80_) determined a 0 and 7 days post challenge (dpc). Cattle were used to test the efficacy of a candidate RVFV subunit vaccines containing a combination of GnGc or Gn alone with or without a booster vaccination and the production of neutralizing antibodies was determined. Significance was determined by two-way ANOVA with Tukey’s multiple comparisons test for adjusted p values using Graph Pad Prism software. The PRNT_80_ titers of GnGc/-, GnGc/GnGc and Gn/Gn groups were significantly different (* *p* value < 0.05) from placebo and -/Gn groups at 0 dpc, and all vaccinated groups were significantly different (* *p* value < 0.01) from the placebo group at 7 dpc.

On the day of challenge, animals that had received the Gn and/or GnGc vaccine candidates on vaccination day 0, independent of booster vaccination, had all developed detectable neutralizing antibody titers (Figure 4). Only the two groups that received a booster vaccination at 21 dpv developed a PRNT_80_ titer of ≥40. At 7 dpc, all animals, including group 4 with the Gn vaccination at 21 dpv, had a detectable PRNT_80_ titer. At 7 dpc, group 1, which received the GnGc/GnGc vaccine, had a PRNT_80_ titer range of 80–320, while group 2, which received two doses of the Gn vaccine, had a PRNT_80_ titer range of 40–320. Treatment group 3, which received a single dose of GnGc/- at 0 dpv, responded with a lower PRNT_80_ titer of only 20–40 at 7 dpc. Interestingly, group 4, which received only one Gn vaccination at 21 dpv, had a PRNT_80_ titer range of 80–640 at 7dpc.

After challenge with virulent RVFV, elevated body temperatures were first detected at 2 days post challenge (dpc), in one animal from group 5, the placebo control group 5, and in one from group 4, the -/Gn group (Supplemental Table S1). The 2 dpc febrile animal in the placebo group 5 was also febrile at 4 and 5 dpc. The other animal in the placebo group did not manifest a febrile response within the first 3 dpc but died with acute clinical signs at 3 dpc. One animal in the single vaccination Gn/Gc group 3 had an elevated temperature at 3 dpc. Blood plasma clinical parameters were monitored over the course of study. Aspartate aminotransferase (AST) activity increased substantially starting at 2 dpc for the placebo group 5 and at 3 dpc for one animal in the single -/Gn dose group 4; the other clinical parameters included in the VetScan Large Animal Profile reagent were within normal range (Supplemental Table S2), except for animals belonging to the single Gn vaccine group 4, which had a slightly elevated gamma glutamyl transferase (51 U/L; normal range 12–48 U/L); at 7 dpc (#14) and 10 dpc (#11).

**Figure 5:**
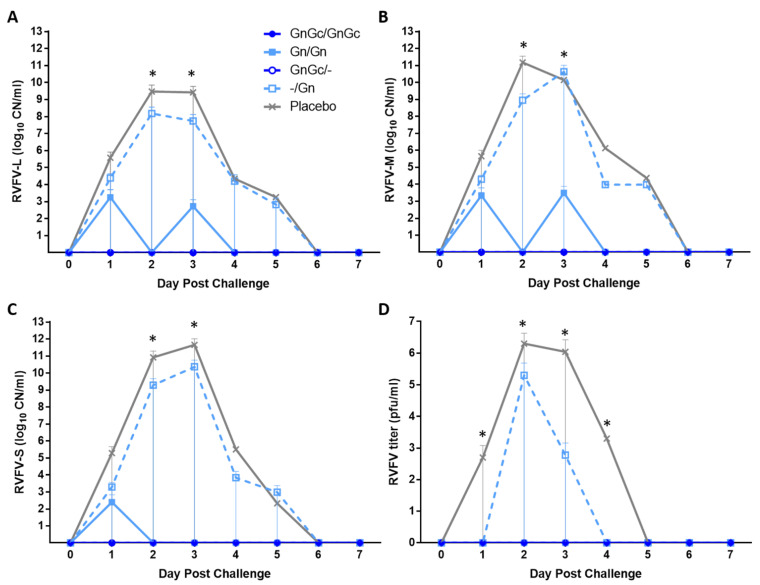
RVFV RNAemia and viremia. Real-time quantitative PCR results are reported in calculated copy number (CN) per ml of sera for (**A**) L, (**B**) M and (**C**) S genomic segments, and (**D**) virus titers are reported as pfu/mL of sera. Mean and standard deviations are shown. Significance was determined by two-way ANOVA with Tukey’s multiple comparisons test for adjusted p values using Graph Pad Prism software. (**A**) The L RNA CN of GnGc/-, GnGc/GnGc and Gn/Gn groups were significantly different (* *p* < 0.05) compared to placebo at 2 dpc; GnGc/- and GnGc/GnGc groups were significantly different (* *p* < 0.05) compared to placebo at 3 dpc. (**B**) The M RNA CN of GnGc/-, GnGc/GnGc and Gn/Gn groups were significantly different (* *p* < 0.05) compared to placebo at 2 dpc; GnGc/-, GnGc/GnGc were significantly different (* *p* < 0.05) compared to placebo and -/Gn at 3 dpc. (**C**) The S RNA CN of GnGc/-, GnGc/GnGc and Gn/Gn groups were significantly different (* *p* < 0.05) compared to placebo at 2 dpc; GnGc/-, GnGc/GnGc and Gn/Gn were significantly different (* *p* < 0.05) compared to placebo and -/Gn at 3 dpc. (**D**) Virus titers of GnGc/-, GnGc/GnGc, Gn/Gn and -/Gn groups were significantly different from placebo at 1 dpc (* *p* < 0.05); GnGc/-, GnGc/GnGc and Gn/Gn were significantly different from placebo and -/Gn at 2 dpc (* p < 0.0001); GnGc/-, GnGc/GnGc and Gn/Gn groups were significantly different from -/Gn (* *p* < 0.05), and GnGc/-, GnGc/GnGc, Gn/Gn and -/Gn groups were significantly different from placebo (* *p* < 0.0001) at 3 dpc; GnGc/-, GnGc/GnGc, Gn/Gn and -/Gn groups were significantly different from placebo (* *p* < 0.0001) at 4 dpc.

None of the GnGc/GnGc (group 1) or GnGc/- (group 3) animals had detectable viral RNA or virus in their serum at any day after RVFV challenge (Figure 5). Animal #10 from group 2 (Gn/Gn vaccination) had low levels of RVFV L segment (1.06 × 10^3^ copy number [CN]) and M segment (6.24 × 10^3^ CN) RNA at 3 dpc, but the RVFV S segment was not detected (Figure 5A–C). The group 4 animals #11 and #14, receiving a single Gn vaccination at 21 dpv, had 2 to 4 logs of detectable RVFV L, M and S RNA at 1 dpc, peaking at 3 dpc with 8 to 10 logs RNA CN. Both animals #12 and #15 of the placebo group 5 also had detectable viral RNA at 1 dpc with 3 to 5 log RNA CN, peaking at 3 dpc with 8 to 11 log RNA CN. The infectious RVF virus was detected in the serum of all animals of groups 4 and 5, which were also clearly positive for RVFV RNA after RVFV challenge. The two animals (#11 and #14) in group 4 had maximal virus titers on 2 dpc with 4 × 10^5^ pfu/mL for #11 and 6 × 10^2^ pfu/mL for #14 (Figure 5D). Animal #11 had detectable virus in serum at 2–3 dpc, while #14 was only viremic at 2 dpc. The placebo group 5 animals, #12 and #15, also had maximal virus titers at 2 dpc, with 4 × 10^5^ pfu/mL for #12 and 3.6 × 10^6^ pfu/mL for #15. Animal #12 was viremic from 1 dpc to 4 dpc. Animal #15 was viremic from 2 dpc to 3 dpc when it died; this animal had the highest serum virus titer of any animal. No virus was detected in the serum from animals in groups 1–3 after challenge.

### 3.3. Pathology, Immunohistochemistry and Virus Loads in Organs

In experimental and natural RVFV infections, the liver is the primary target organ for pathology, thus for all animals included in this study, the gross and histological changes in the liver were summarized and characterized. Histological liver lesions were scored following a previously established scoring scheme (28). Virus titration assays were completed on homogenates of kidney, liver and spleen collected at the time of necropsy. The gross pathology for animal #15 from the placebo group 5 that died 3 dpc showed gross liver lesions consistent with acute RVF hepatopathy, multifocal necrotic foci and hemorrhage within all liver lobes, diffuse pallor of the liver parenchyma and rounding of the lobe margins (hepatic swelling). Cultivatable virus was only recovered from fresh tissue from animal #15 which died on 3 dpc with severe acute RVF disease. The liver, kidney and spleen had titers of 6.3 × 10^5^, 2 × 10^5^ and 4 × 10^3^ pfu/mL, respectively. All other animals euthanized on 7 dpc and 10 dpc were negative for infectious virus in these tissues.

Table 1 summarizes the histopathology analysis of the liver and the presence of viral antigen (IHC), viral RNA (RT-qPCR) and infectious virus of all animals enrolled in the study. Animals in groups 1–3 showed none to minimal inflammatory infiltrates within the liver (liver scores < 1) and were negative for the presence of viral RNA, viral antigen and infectious virus. The two animals in each groups 4 and 5 had liver histopathology scores ≥2 representative of mild to moderate liver lesions, with one animal from group 4 and both animals from group 5 being positive for viral RNA and one (#15) being positive for the presence of viral RNA, viral antigen and infectious virus.

**Figure 6:**
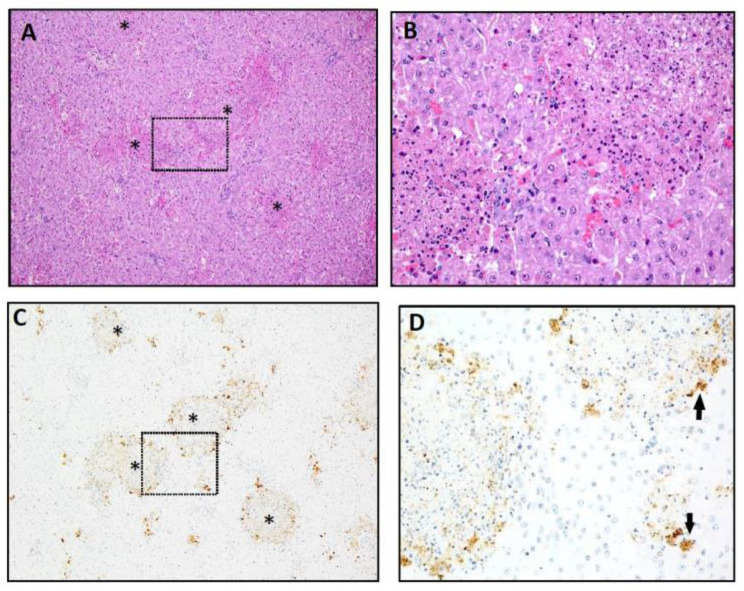
Hepatic lesions of a placebo vaccinated animal (#15. Group 5) that died of acute Rift Valley fever at 3 dpc. The histopathology (H) score was 4 and is representative of severe RVFV hepatic lesions. (**A**) 100× magnification of a hematoxylin and eosin stain (H&E) of liver with severe multifocal necrosis and hemorrhage. Larger lesions are marked (*). The broken line outlines the area magnified in B (200×). (**B**) Larger foci of acute hepatic necrosis consisting of hypereosinophilic and karyorrhectic debris; inflammation is minimal. (**C**) 100× magnification of IHC for viral antigen in the same section as A is strongly positive for RVFV antigen (brown) in the cytoplasm of hepatocytes rare macrophages and cellular debris. The broken line outlines the area magnified in D (200×). (**D**) Viral antigen is most prevalent in necrotic hepatocytes at lesion periphery and in smaller foci of necrotic hepatocytes (arrows).

Histopathological findings of severe multifocal necrosis (histopathology score of 4) accompanied by prominent IHC labeling for RVFV antigen (Figure 6) confirm acute RVFV hepatitis in animal #15. Figure 6A is a Hematoxylin and Eosin (H&E) stained slide of liver demonstrating disruption of the hepatic lobules with multifocal to coalescing foci of hepatic necrosis involving greater than 20% of the hepatic parenchyma (Figure 6A). Necrotic foci ranged in size from a single cell to greater than 1–2 mm in diameter. Larger foci were characterized by central areas of eosinophilic cellular debris, fibrin and karyorrhectic debris, occasionally accompanied by hemorrhage. Admixed and bordering these foci were swollen, hypereosinophilic and necrotic hepatocytes and minor infiltrates of macrophages and lymphocytes (Figure 6B). Smaller foci, including aggregates or individual hepatocytes undergoing cell death, were scattered throughout the parenchyma and the remaining hepatocytes were swollen and lightly vacuolated. Necrotic foci were positive for viral antigen by IHC with the typical pattern of the strongest labeling at the periphery of the lesions (Figure 6C,D).

**Figure 7:**
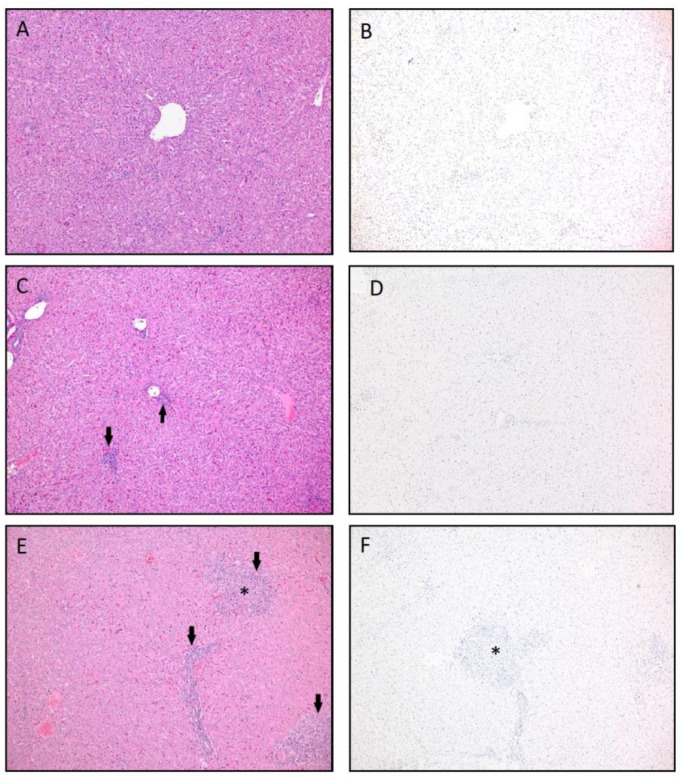
Liver histopathology and viral antigen immunohistochemistry (IHC) representative of absent to minimal to moderate hepatic inflammation in vaccinated animals. (**A**) Hematoxylin and eosin stain (H&E) of normal liver. H score was 0 = no lesions (Group 3, Animal #3, 10 dpc). (**B**) No viral antigen was present by IHC (Group 3, Animal #3, 10 dpc). (**C**) H&E of the liver with low numbers of periportal lymphocytes and plasma cells (arrows). H score was 1 = minimal inflammation (Group 2, Animal #9, 10 dpc). (**D**) The tissue was also negative for viral antigen by IHC (Group 2, Animal #9, 10 dpc). (**E**) H&E of multifocal inflammation in portal tracts (arrow) as well as a focus of inflammation with hepatocyte loss (*). H score was 2.5 = moderate inflammation (Group 4, Animal #14, 7 dpc). (**F**) IHC was negative for viral antigen (Group 4, Animal #14, 7 dpc). 100× magnification. The H score is the hepatic histopathology lesion score based on the lesion character, amount of hepatic parenchyma affected and lesion severity on a scale from 0 (no lesions) to 4 (severe lesions_ (see Table 1)).

The remaining animals in the study were necropsied at 7 dpc (#1, 2, 4, 5, 6, 12, 14) and 10 dpc (#3, 9, 10, 11) following virus clearance, and no gross liver lesions were appreciated. Significant histological lesions consistent with chronic RVFV infection were not present in liver in the H&E sections from the group 1, group 2 and group 3 animals. Chronic RVFV lesions were seen in animals receiving a single Gn dose (#11 and #14) and those within the placebo group (#12), groups 4 and 5, respectively. Figure 7 shows a panel of images of the H&E and IHC stained liver slides representative of chronic RVFV histopathology observed on 7 dpc and 10 dpc in groups 4 and 5 animals. Figure 7A shows a histologically normal liver (animal #3; histopathology score 0), 7C represents mild periportal hepatic inflammation (animal #9, histopathology score 1) and 7E has moderate periportal inflammation and hepatic necrosis with moderate inflammatory cell infiltrates indicative of more virus damage to the liver (animal #14; histopathology score 2.5). All livers from the 10 dpc necropsy re negative for viral antigen by IHC (Figure 7B,D,F), results that are consistent with virus isolation and RT-qPCR results (Table 1). Significant histological lesions consistent with chronic RVFV infection were not noted liver in H&E sections from group 1, groups and group 3 animals. Chronic RVFV lesions were seen in animals receiving a single Gn dose (#11 and #14) and those within the placebo group (#12) (Table 1).

## 4. Discussion

The extended geographical range of mosquito species capable of transmitting virulent pathogens is of great concern and has led to the emergence of animal and human arboviruses in new territories in the past decades [2,29]. Rift Valley fever is one of these arboviral diseases which was detected for the first time outside its traditional boundaries in Africa when it was found in the Arabian Peninsula in 2003 [2,29]. Additional outbreaks occurred in South Africa (2008–2010) [30] and reemergence in 2018 [31]. Niger and Uganda were free of reported human RVFV cases for many years, but the disease has re-occurred there in 2016 [32,33]. Similarly, ruminant and human RVFV cases were first reported in Mayotte in 2007, and RVF cases re-occurred in 2018–2019 [34]. While RVF outbreaks are notable, it should be noted that low levels of RVFV circulates among mosquito vectors and susceptible mammalian species in endemic regions between epidemics [35]. Thus, RVF continues to be of importance for both, public and veterinary health. In addition, RVF has negative socio-economic effects in endemic countries [36], thus effective mitigation strategies are urgently needed. These mitigation strategies include mosquito control and vaccination of susceptible domestic ruminants. The ideal RVFV vaccine should be cheap, safe and efficacious vaccine and able to induce long-term immunity with DIVA compatibility, the latter being especially important for non-endemic countries. Unfortunately, such a vaccine is currently not available. Therefore, we developed a DIVA-compatible subunit RVFV vaccine that has been proven safe and efficacious in sheep [15,21]. The present study was designed to provide evaluation of the subunit RVFV vaccine approach in cattle and to evaluate different vaccination regimens and formulations.

Due to space limitations in our BSL-3Ag containment, the number of calves assigned to the various treatment groups was rather small. In our sheep RVF model, we utilized a combination of the recombinant Gn and Gc glycoproteins, produced in insect cells, in the vaccine formulation. Subsequent studies in a mouse model of RVF demonstrated that vaccination with the recombinant Gn alone might be sufficient for protection against virulent RVFV challenge (unpublished data). In addition, it has been demonstrated by others that the ectodomain of Gn is effective in protecting sheep from RVFV challenge [37]. Consequently, in this study using cattle, animals were administered a combination of Gn and Gc or Gn alone in either a single- or two-dose regimen in order to determine whether Gn vaccination alone was sufficient for protection of cattle against RVF. In addition, since vaccinations of livestock are often administered during a RVF outbreak, the ability to provide two doses of the vaccine may be limited. Since the time between vaccination and RVFV exposure may be short, two additional vaccination regimens were tested to evaluate the time between vaccination and the onset of protective immunity. The first vaccination approach was a single-dose vaccination with a Gn and Gc combination administered 35 days before challenge (group 3) and the second vaccination approach was a single vaccination 14 days before challenge (group 4). The latter time point for vaccination was selected to determine if any protection could be afforded within 2 weeks after vaccination.

Calves vaccinated with Gn or GnGc exhibited substantial antibody response to Gn when administered in a two-dose regimen (Figure 2A). Even a single-dose Gn or GnGc vaccination elicited a significant in duction of Gn-specific antibodies. Overall, the calves’ antibody responses to the Gc antigen were not as robust as the response to Gn (Figure 2B). In fact, there was also evidence of a potential cross reaction response to Gc antigen after Gn-immunized calves received a second dose of the Gn only vaccine. Presumably, this is due to the fact that the recombinant Gn and Gc glycoproteins have a His-tag attached to their N-terminal domains for protein purification, which was not removed before immunization, or due to common contaminates in the SF9 insect cells used to produce the recombinant proteins. In contrast, a single dose of GnGc did not produce an antibody response to Gc. This is consistent with our previous observation of Gc not being as immunogenic when compared to Gn [38].

To further evaluate the serological response in vaccinated and RVFV challenged animals, a FMIA specific for the RVFV N and Gn antigens [27] and a PRNT_80_ assays were used to evaluate the sera post challenge. The FMIA simultaneously detects antibodies to the RVFV N and Gn proteins, thus determining the DIVA compatibility of the subunit RVFV vaccine approach. Antibodies to N were only detected above background at 7 dpc using the FMIA in the placebo vaccine/challenge control group 5 and in the -/Gn group 4 one dose regimen (one 21 dpv administration) animals at 7 dpc (Figure 3A). Only the vaccinated animals and not the animals receiving placebo had antibodies to Gn at the time of challenge at 0 dpc (Figure 3B). Post RVFV challenge, there was a significant increase in antibodies specific for Gn in all vaccinated animals except the placebo group (Figure 3B). This indicates that vaccination with a single Gn dose (group 4) even 14 days before infection may have some benefits increasing antibody responses to -/Gn. This was supported by the increase in PRNT_80_ titer for the-/ Gn single dose at 7 dpc (Figure 4). Surprisingly, the two-dose GnGc group 1 animals had only a moderate neutralizing antibody response. This is most likely due to a rapid immune-mediated clearance of inoculum without efficient virus replication. Variations in animal serological responses to RVFV infection and the impact of immunization have been described previously [28].

In the present study, calves were challenged with a RVFV strain which was shown to be highly virulent for cattle and sheep [28]. Infection of unvaccinated control cattle with the RVFV Kenya-128B-15 strain resulted in clinical disease characteristic of RVFV infection and in death in one of these animals in group 5. All animals in the four vaccine groups were protected from the development of acute Rift Valley fever symptoms and all survived the challenge. Viremia and RNAemia (virus isolation and positive RT-qPCR for all three RVFV genes) was only detected in animals administered the single dose of -/Gn vaccine 14 days prior to challenge and the unvaccinated control animals. Viremia in this -/Gn vaccine group 4 was detected on 2 dpc and 3 dpc following challenge (1 dpc) and was shorter in duration when compared to that of the unvaccinated challenge controls, suggestive of partial immune protection after a single dose vaccination only 14 days prior to virulent RVFV challenge. In contrast, the Gn two-dose prime boost regimen did provide full protection from RVFV infection and disease. The presence of neutralizing antibodies is considered a good corelate of protection from RVFV infection with titers ≥1:40 considered being protective [9]. The neutralizing antibody responses are known to be directed towards Gn and Gc [24,39,40]. At the time of design of the present study, we had preliminary mouse data that suggested Gn alone would be sufficient for protection (unpublished data); this is supported by the present cattle study. This finding is consistent with a RVFV Gn head domain vaccine study in sheep [41]. The other single dose group (group 3) in the present study was administered the GnGc vaccine 35 days prior to RVFV challenge and did not exhibit viremia and showed only transient RNAemia only for the RVFV L and M genes; however, one animal in this group had a slightly elevated body temperature for one day post challenge. Thus, this GnGc/- single dose regimen 35 days before infection provided good protection against clinical RVF and viremia. Although the number of animals in this study is small, the presented data demonstrates that a single dose of the Gn or GnGc vaccine administered 35 or 14 days prior to virulent RVFV challenge, respectively, may offer at least some protection against the development of acute RVF; however, a two-dose prime boost regimen provides the foundation needed for the development of sterile immunity and protection from clinical RVF.

## 5. Conclusions

The RVFV subunit Gn and GnGc vaccine formulations administered in a prime boost regimen provided excellent efficacy and sterile immunity in cattle. A single dose Gn or GnGc vaccine regimen provided sufficient protection and immunity when administered 35 days before challenge, but a single dose Gn provided less protection when administered 14 days before challenge indicating two weeks is not sufficient time for the development of a protective immune response against RVFV infection. Further studies are needed to confirm these results in a larger study, and to address onset and duration of immunity as well as variations in vaccine responses in cattle.

## Figures and Tables

**Table 1:** Histopathology scores, presence of RVFV antigen, RVFV RNA and infectious virus in the liver.

Group	Calf ID#	DPC *	Histo-Pathology Score **	RT-qPCR ***	IHC	Virus Titer
1. GnGc/GnGc	1	7	0	--	--	--
	2	7	0	--	--	--
	5	7	0	--	--	--
2. Gn/Gn	6	7	0	--	--	--
	9	10	1	--	--	--
	10	10	1	--	--	--
3. GnGc/-	3	10	0	--	--	--
	4	7	0	--	--	--
4. -/Gn	11	10	2	--	--	--
	14	7	2.5	POS	--	--
5. Placebo	12	7	3	POS	--	--
	15	3	4	POS	POS	9.0 × 10^5^

* DPC is days post challenge. ** Scored as per (28). *** Positive for RVFV L, M and S gene detection from formalin fixed paraffin embedded tissues.

## Data Availability

The data reported in this study will be archive in the U.S. National Agriculture Library according to USDA polices.

## Supplementary Materials

The following are available online at https://www.mdpi.com/article/10.3390/vaccines9070748/s1, Table S1: Body temperatures after Rift Valley fever virus challenge; Table S2: Aspartate aminotransferase (AST) levels after Rift Valley fever virus challenge.
