# Supplementary material for: Preliminary Evaluation of a Recombinant Rift Valley Fever Virus Glycoprotein Subunit Vaccine Providing Full Protection against Heterologous Virulent Challenge in Cattle
Source: Vaccines (Basel). 2021 Jul 6;9(7):748. doi: 10.3390/vaccines9070748 (PMC8310273; doi:10.3390/vaccines9070748)
Supplement: Supplementary file 1 [file vaccines-09-00748-s001.zip › vaccines-1253454-supplementary.pdf]

Table S1. Body temperatures after Rift Valley fever virus challenge. Yellow shades indicated values above normal range for bovine. Black shades indicates animals that have died or have been euthanized.

Animal ID	Vaccine Group	Body Temperature								
		0 dpc	1 dpc	2 dpc	3 dpc	4 dpc	5 dpc	6 dpc	7 dpc	10 dpc
1	GnGc/GnGc	38.8	39.0	39.3	38.6	38.6	38.2	38.8	38.5	
2	GnGc/GnGc	38.8	39.2	39.0	39.7	39.3	39.3	39.3	38.7	
5	GnGc/GnGc	37.3	38.8	38.9	38.8	38.8	39.2	38.7	38.7	
6	Gn/Gn	38.4	38.9	38.6	38.7	38.3	38.7	38.2	38.6	
9	Gn/Gn	39.1	39.1	39.2	38.9	39.3	39.1	39.1	39.2	38.8
10	Gn/Gn	38.6	38.7	38.9	39.1	38.7	38.7	38.6	38.7	38.7
3	Gn/Gc (0 dpv)	39.9	39.0	38.8	38.7	39.4	38.5	38.8	39.3	38.6
4	Gn/Gc (0 dpv)	38.7	39.3	38.8	40.6	38.4	39.4	39.4	38.4	
11	Gn (21 dpv)	39.1	39.1	39.5	39.5	38.6	38.3	38.6	38.6	37.9
14	Gn (21 dpv)	38.8	39.2	41.3	39.7	39.1	39.2	39.1	38.6	
12	Placebo	38.7	39.7	41.0	39.8	40.2	40.2	39.9	39.3	
15	Placebo	38.3	38.4	38.4	38.4					

Table S2. Aspartate aminotransferase (AST) levels after Rift Valley fever virus challenge. Yellow shades indicated values above normal range for bovine. Normal range for bovine 66-211 U/L. Black shades indicates animals that have died or been euthanized.

Animal ID	Vaccine Group	Aspartate aminotransferase U/L								
		0 dpc	1 dpc	2 dpc	3 dpc	4 dpc	5 dpc	6 dpc	7 dpc	10 dpc
1	Gn/Gc	75	76	63	71	NA	102	90	100	
2	Gn/Gc	76	74	83	83	72	74	90	93	
5	Gn/Gc	90	73	65	64	61	57	55	63	
6	Gn/Gn	83	83	83	107	93	91	90	95	
9	Gn/Gn	98	92	91	97	95	105	102	100	122
10	Gn/Gn	59	59	59	60	58	55	55	59	100
3	GnGc/-	63	64	63	67	70	76	89	74	80
4	GnGc/-	54	50	50	52	51	55	63	76	
11	-/Gn	68	NA	124	250	148	119	258	268	168
14	-/Gn	84	72	108	108	89	98	92	88	
12	Placebo	65	67	240	457	249	149	110	89	
15	Placebo	71	69	406	1709					
